# Association between Baseline Cognitive Score and Postoperative Delirium in Parkinson's Disease Patients following Deep Brain Stimulation Surgery

**DOI:** 10.1155/2022/9755129

**Published:** 2022-07-11

**Authors:** Yongde Zhou, Ting Fan, Yu Ma, Jian Ding, Jianfeng Yu, Yao Chen, Cuiping Yu, Rongsong Zhou, Baoguo Wang, Chengmei Shi

**Affiliations:** ^1^Department of Anesthesiology, Tsinghua University Yuquan Hospital, Beijing 100040, China; ^2^Department of Neurosurgery, Tsinghua University Yuquan Hospital, Beijing 100040, China; ^3^Department of Anesthesiology, Sanbo Brain Hospital Capital Medical University, Beijing 100093, China; ^4^Department of Anesthesiology, Peking University Third Hospital, Beijing 100191, China

## Abstract

**Background:**

Deep brain stimulation of the subthalamic nuclei (STN-DBS) is a standard treatment option for advanced Parkinson's disease (PD) patients. Delirium following DBS electrode implantation is common, by several studies, and cognitive impairment is a risk factor for developing postoperative delirium (POD). This prospective observational study was conducted to identify whether preoperative baseline cognitive status has an association with POD in PD patients undergoing DBS surgery.

**Methods:**

Preoperatively, neuropsychiatric and neuropsychological assessments of the patients were performed including clinical dementia rating (CDR) score, instrumental activities of daily living (IADL) score, mini-mental state exam (MMSE) score, Montreal cognitive assessment (MoCA) score, Hamilton anxiety (HAMA) and Hamilton depression (HAMD) scores, and numerical cancellation test. POD was identified by the confusion assessment method (CAM) twice per day on postoperative day 1 until discharge.

**Results:**

Twenty-seven (21.6%) of 125 patients developed POD. Among the variables screened, age, CDR score, MMSE score, and HAMA score were indicated to be independent influence factors of POD. The cutoff score, AUC, sensitivity, and specificity of age, CDR score, MMSE score, and HAMA score associated with POD was 58.5, 0.751, 92.6%, 52.0%; 0.5, 0.848, 77.8%, 91.8%; 27.5, 0.827, 88.9%, 62.2%; and 12.5, 0.706, 85.2%, 54.1%, respectively.

**Conclusions:**

We observed age, CDR score, MMSE score, and HAMA score were independent influence factors of POD in PD patients who received DBS. It is necessary to assess the cognitive status of PD patients before surgery to identify high-risk patients.

## 1. Introduction

Parkinson's disease (PD) is a common neurodegenerative disease, with the main clinical symptoms being static tremors, muscle rigidity, and bradykinesia [1]. Deep brain stimulation of the subthalamic nuclei (STN-DBS) is a standard treatment option for advanced PD patients. Bilateral STN-DBS not only improves motor symptoms but also a variety of nonmotor symptoms [2–4], as well as health-related quality of life [2, 5]. DBS could also reduce the levodopa medication dose and ameliorate the side effects associated with levodopa therapy [4].

However, delirium is one of the most common neuropsychiatric complications following DBS surgery [6], occurring in approximately 22–42.6% of patients [7,8]. Postoperative delirium (POD) is an acute disorder of attention and cognition in elderly people that is common, serious, costly, under-recognized, and often fatal [9]. POD has been independently associated with worsened clinical outcomes, increased costs, and increased mortality in patients [9]. For patients with Parkinson's, delirium is an increased risk factor for developing dementia, having a more severe motor impairment, and death [10].

Cognitive impairment is a risk factor for the development of POD [11–13]. For example, preoperative screening of MMSE [14], MoCA [15], depression [16], and anxiety [17] was associated with POD.

This prospective observational study was conducted to identify whether preoperative baseline cognitive status has an association with POD in PD patients undergoing DBS surgery.

## 2. Methods

### 2.1. Study Design and Clinical Assessment

This was a prospective, cohort study. The research proposal has been approved by the Ethics Committee of Yuquan Hospital of Tsinghua University (20190014). All patients enrolled signed informed consent. The clinical trial registration was completed before the first patient is enrolled (https://www.chictr.org.cn, ChiCTR1900027210).

### 2.2. Subject

A total of 128 consecutive PD patients from Tsinghua University Yuquan Hospital treated with bilateral STN-DBS were screened at baseline. All patients were diagnosed with PD according to the UK Brain Bank criteria [18]. Bilateral STN-DBS treatment was initiated according to the Movement Disorders Society guidelines [19].

According to Chinese deep brain stimulation therapy for Parkinson's disease expert consensus (Second Edition) [20], the inclusion criteria for performing DBS surgery are as follows: primary PD, hereditary PD or various genotypes PD, responds well to compound levodopa; drug efficacy has decreased significantly or obvious motor complications affect the patient's quality of life; adverse drug reactions that cannot be tolerated and affect the efficacy of drugs; and there are tremors that cannot be controlled by drugs. Contraindications for performing DBS surgery are as follows: significant cognitive impairment; severe (refractory) depression, anxiety, schizophrenia, and other mental diseases; and medical comorbidities that affect surgery or survival.

Preoperatively, neuropsychiatric and neuropsychological assessments of the patients were performed including the CDR score, IADL score, MMSE score, MoCA score, Hamilton anxiety (HAMA), Hamilton depression (HAMD) score, and numerical cancellation test.

Baseline information such as age, sex, body mass index, the highest level of education, American Society of Anesthesiologists (ASA) functional status, and preoperative complications was recorded.

### 2.3. Anesthesia Method

The general anesthesia and surgery were operated by a team to avoid interfering factors. After the patients were transferred to the operating room, the electrocardiograph, noninvasive blood pressure, heart rate, saturation of pulse oximetry, and bispectral index (BIS) were monitored. 2 ml venous blood was collected when the peripheral vein was accessed. The induction drugs were sufentanil 0.3 *μ*g/kg, propofol 1.0–2.0 mg/kg, etomidate 0.2-0.3 mg/kg, and cisatracurium 0.2 mg/kg. After induction, a 7.5^#^ (female) or 8.0^#^ (male) endotracheal tube was intubated.

In the anesthesia maintenance stage, patients randomly received total intravenous anesthesia (TIVA) or combined intravenous and inhalation anesthesia (CIIA). The anesthetics for TIVA were propofol (4.0–8.0 mg kg^−1^ h^−1^) and remifentanil (0.1–0.4 *μ*g kg^−1^ h^−1^), and sevoflurane (1–1.5%), remifentanil (0.1–0.4 *μ*g kg^−1^ h^−1^) were used in CIIA. All patients received BIS (BIS 40–60) monitor to adjust anesthesia depth [21]. Vasoactive drugs were used to maintain hemodynamic stability if necessary. After surgery, all the patients received the same analgesic strategy (sufentanil 2 *μ*g/kg + dexmedetomidine 2.3 *μ*g/kg diluted to 100 ml), the background infusion rate was 2 mL/h, the dosage of PCA was 0.5 mL, and the locking time was 15 min.

The anesthesia time, operation time, intraoperative fluid volume, hypotension, bradycardia, and other side effects were recorded.

### 2.4. Operation

All patients included in the study were diagnosed with PD and met DBS indications.

Patients underwent two surgeries in this study. In the first surgery, patients underwent stereotactic implantation of DBS electrode in the subthalamic nucleus (STN). The anesthesia method usually was local anesthesia with minimal sedation. They then underwent imaging examination to confirm the place of the electrodes.

The second surgery was conducted after the imaging confirmation and the DBS batteries and leads were placed. The second surgery was performed under general anesthesia, the DBS generator was implanted in the subclavicular region, and the extension wires were tunneled through the neck and connected to the DBS electrode. The patient returned to the ward after extubation.

All the patients received an assessment of the unified Parkinson's disease rating scale (UPDRS) score three times and in two phases: preoperative, 2 weeks after surgery, and 6 months after surgery and medication on and off phase. The levodopa equivalent daily doses (levodopa equivalent daily dose) before, 2 weeks, and 6 months after surgery were also recorded. The stimulation generator was switched on 2 weeks after DBS surgery. The postoperative followup was operated 6 months after DBS surgery.

### 2.5. Clinical Assessment

POD was identified by the confusion assessment method (CAM) [9] which could be used for medical staff, caregivers, and family members. The CAM was administered twice per day on postoperative day 1 until discharge. POD assessment is generally divided into two steps: first, use Richmond agitation-sedation score (RASS) to evaluate patients' consciousness, and second, evaluate the content of consciousness. If someone's RASS score was −4 or −5, then the patient could not be evaluated. The whole process needed about 5 min, including the following 4 questions: (1) acute onset fluctuated mental status; (2) attention deficit; (3) altered level of consciousness (RASS score); and (4) confusion. If 1 + 2 + 3/4 is matched, delirium was diagnosed [22].

The assessment of POD was performed twice a day between 7 : 00 am and 7 : 00 pm after the surgery until discharge. The occurrence of POD was assessed by using the CAM [9] and the severity of the POD was assessed by using the memorial delirium assessment scale (MDAS) [23].

Three patients were excluded due to the second operation. Therefore, 3 patients were excluded from further analysis. Finally, 125 PD patients were analyzed.

### 2.6. Statistical Analysis

SPSS 26.0 software was used for data analysis. The Kolmogorov–Smirnov test was used first to test the normality of all of the variables. Mean ± standard deviation ($\bar{x}$±*s*) was used for statistical description, and an independent sample *t*-test or one-way analysis was used for the normal distribution variables comparisons between groups. Median (interquartile spacing) was used for statistical description, and the Kruskal–Wallis test was used for abnormal distribution variables comparisons between groups. Percentages were used for statistical description, and the *χ*^2^ or Fisher exact test was used for counting data. A binary logistic regression was used to estimate the odds ratio (OR) of maintaining independence. *P* < 0.05 was considered statistically significant.

## 3. Results

A total of 128 patients were screened, and 3 patients were excluded because of the second operation. Thus, 125 patients completed both preoperative and postoperative assessments and were entered into the final analysis. The symptoms were improved after DBS surgery. The UPDRS part II and part III score and levodopa equivalent daily dose (LEDD) were significantly decreasing than preoperative. There was no serve complication during the perioperative period (Table 1).

Patients were divided into two groups according to the occurrence of POD: POD group (27 cases) and non-POD group (98 cases).

### 3.1. Clinical Data of Patients with POD

Twenty-seven patients were diagnosed with POD, and the incidence of POD was 21.6% (27/125). All 27 patients developed POD on the first postoperative day. Delirium disappeared on the second day after surgery in 19 patients (70.37%).

Among 27 patients with POD, high activity delirium accounted for 66.67% (18/27), and mixed and low activity types were 18.52% (5/27) and 14.81% (4/27), respectively.

### 3.2. Baseline Characteristics of POD and Non-POD Group

There were no differences between POD and non-POD groups on genders, body mass index (BMI), ASA status, preoperative complications, LEDD, the rate of receiving dopamine receptor agonists and MAO inhibitors preoperative, operation time, and general anesthesia maintenance method. However, the age of patients in the POD group was significantly elder than in the non-POD group. The years of education in the POD group were significantly longer than in the non-POD group. The anesthesia time of the POD group was significantly longer than the non-POD group (Table 2).

### 3.3. Comparison of Preoperative Cognitive Status between the Two Groups

There were no differences between POD and non-POD groups on the correct elimination and elimination index. However, there was a significant difference between POD and non-POD groups on the CDR score, IADL score, MMSE score, MoCA score, HAMA and HAMD score, missing elimination, incorrect elimination, and elimination time (Table 3).

### 3.4. Univariate and Multivariate Logistic Regressions

Univariate and multivariate logistic regressions were performed to identify the potential risk factors of POD. Each variable was screened using the univariate regression and the variables with *P* < 0.05were selected for multivariate logistic regression. Those variables with *P* < 0.05in the multivariate regression were defined as having an association with POD. The odds ratio (OR) and 95% confidence interval (95% CI) were used to illustrate the predictive power of certain characters.

Among the variables screened, age, CDR score, MMSE score, and HAMA score were indicated to be independent influence factors of POD (Table 4).

### 3.5. Diagnostic Analysis of Indicators for the Prediction of POD

The AUC, cutoff, sensitivity, specificity, and Youden index of independent influence factors of POD are given in Table 5.

A receiver operating characteristic (ROC) curve was used to determine the optimal cutoff score for the diagnosis of POD. The optimal score was calculated according to the Youden index (maximum of (sensitivity + specificity−1)) [24]. The total area under the curve (AUC), the sensitivity, and the specificity were all used for this determination.

**Figure 1:**
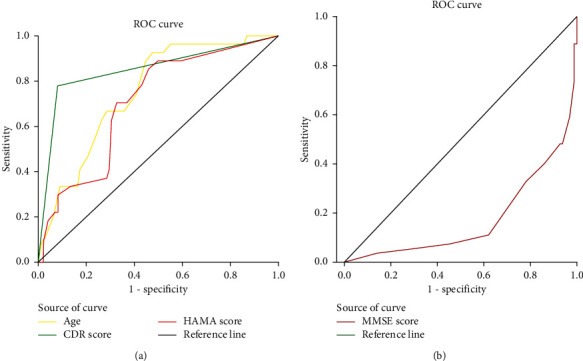
(a) ROC analysis used for the determination of the diagnostic sensitivity and specificity of the preoperative optimum cutoff score of age, CDR score, and HAMA score. The probability of the POD increases with the increase in age, CDR score, and HAMA score. (b) ROC analysis used for the determination of the diagnostic sensitivity and specificity of the preoperative optimum cutoff score of the MMSE score. The probability of the POD decreases with the increase of the MMSE scores.

We obtained 58.5 as the optimal cutoff score of age associated with POD. This cutoff score of age led to a sensitivity of 92.6% and a specificity of 52.0% for the association with POD. The AUC was 0.751 (95% CI: 0.657–0.844, *P* < 0.001) (Table 5, Figure 1(a)). We obtained 0.5 as the optimal cutoff score of the CDR score associated with POD. This cutoff score of the CDR score led to a sensitivity of 77.8% and a specificity of 91.8% for the association with POD. The AUC was 0.848 (95% CI: 0.750–0.946, *P* < 0.001) (Table 5, Figure 1(a)). We obtained 12.5 as the optimal cutoff score of the HAMA score associated with POD. This cutoff score of HAMA score led to a sensitivity of 85.2% and a specificity of 54.1% for the association with POD. The AUC was 0.706 (95% CI: 0.601–0.811, *P* < 0.001) (Table 5, Figure 1(a)). We obtained 27.5 as the optimal cutoff score of the MMSE score associated with POD. This cutoff score of MMSE score led to a sensitivity of 88.9% and a specificity of 62.2% for the association with POD. The AUC was 0.827 (95% CI: 0.733–0.920, *P* < 0.001) (Table 5, Figure 1(b)).

## 4. Discussion

Parkinson's disease is a common neurodegenerative disease, which is more common in the elderly. The prevalence of PD in people over 65 years of age is approximately 1.0–3.0% [25]. Deep brain stimulation of the subthalamic nuclei (STN-DBS) is a standard treatment option for advanced PD [19].

With the progress of population aging, more and more PD patients are expected to receive DBS surgery. So, perioperative optimization management of PD patients should attract the attention of anesthesiologists.

POD is an acute neuropsychiatric syndrome after surgery that is associated with an altered level of consciousness, confusion, and impaired attention [26, 27]. POD in elder adults has been associated with both short-term and long-term adverse consequences [9], prolonged hospital stays [28], higher costs per patient [9, 29], and an eight-fold increased risk of future dementia [30]. Fortunately, delirium may be preventable in one-third of cases [31, 32]. Therefore, it is important to identify high-risk patients and optimize perioperative management to reduce the risk of POD.

The prevalence of POD varies from 5.1 to 52.2% with different types of surgery [9]. Patients with PD are at an increased risk for delirium which may be underdiagnosed due to phenomenological overlap between delirium and chronic neuropsychiatric features of PD or side effects of dopaminergic medication [33]. Both Sakai's [34] and Pan's [35] studies showed that PD is an independent risk factor for POD. Oichi [36] reported that POD was more common in patients with PD (30.3%) than in controls (4.3%) following spinal surgery. Carlson's [37] study showed that POD following implantation of DBS electrodes was common (22% of patients). In this study, the incidence of POD in PD patients following DBS was 21.6%.

As a neurodegenerative disease, the previous focus on PD was mainly on motor function. However, cognitive impairment is a common nonmotor symptom of PD [38]. 80% of PD patients reported experiencing dementia after 8 years [39].

In this study, all patients received a preoperative cognitive examination by a specialized psychiatrist including a CDR score, IADL score, MMSE score, MoCA score, HAMA and HAMD score, and numerical cancellation test. Among them, age, CDR score, MMSE score, and HAMA score were independent influencing factors of POD.

At present, age as an important independent risk factor for POD has become a consensus in the field of neurocognitive research [40, 41]. In this study, we also found that age is an independent risk factor for POD.

This study showed that PD patients had been combined with cognitive impairment before surgery. With an assessment of CDR, a score above 0.5 is considered suspicious dementia [42]. In this study, suspected dementia was found in 21.6% of patients. With an assessment of MMSE and MoCA, cognitive function was impaired in 28.8% and 73.6% of patients, respectively. The cognitive impairment above all indicated that these patients were a susceptible population. According to Chinese deep brain stimulation therapy for Parkinson's disease expert consensus (Second Edition) [20], patients with mild cognitive impairments were included, but patients with mild dementia were excluded from this study.

Khan et. al [43] also confirmed that cognitive deficits are predictive of POD and mortality following TAVI and emphasized the value of screening for geriatric risk factors before TAVI by demonstrating that screening increased the identification of at-risk patients.

This study also showed that PD patients had been combined with emotional abnormalities before surgery. With the assessment of HAMA and HAMD scores, 27.2% were likely to be anxious, 10.4% were anxious, 35.2% of patients were mildly depressed, and 6.4% were moderately depressed.

Depression and anxiety are among the most important nonmotor signs of PD [44]. There are close interactions between depression, anxiety, and PD itself. Some of these symptoms are induced by dopaminergic medications, while others rely on the disease pathophysiology itself [45–47]. Widespread dysfunction of the limbic system has been observed in PD patients [44].

PD-associated depression and anxiety are linked with many anatomical changes within the limbic system [44, 48–50]. Some studies showed the atrophic temporal cortex, particularly the amygdala and hippocampus, in PD patients could participate in mood/emotion learning deficits [51–53]. Anita's study also showed limbic systems were identified by neuroimaging as putative substrates for delirium which support cognitive functions [54].

This study also suggested that the preoperative anxiety score was an independent risk factor of POD in PD patients. Although there was a statistical difference in preoperative depression score between the two groups, the final logistics analysis only suggested that the preoperative anxiety score was an independent influencing factor of POD. In the future, a large sample study needs to confirm the relationship between preoperative depression and POD.

In this study, age, CDR score, MMSE score, and HAMA score were independent influence factors of POD in PD patients who received DBS. The cutoff value was 58.5, 0.5, 27.5, and 12.5, respectively. So, if the PD patients are older than 58.5, CDR score higher than 0.5, MMSE score lower than 27.5, and HAMA score higher than 12.5 before DBS, it is necessary to pay attention to the risk of POD.

In summary, delirium may occur more frequently in PD patients. In this study, age, CDR score, MMSE score, and HAMA score were independent influence factors of POD in PD patients who received DBS. This study highlights that it is necessary to assess the cognitive status of PD patients before surgery to identify high-risk patients. Then, advanced interference treatment would be beneficial to reduce the prevalence of POD, or we could improve the outcomes of PD patients with POD through early identification and treatment.

## Figures and Tables

**Table 1:** Comparison of UPDRS score and LEDD before and after DBS surgery.

Variable	Phase	Preoperative	2 weeks postoperative	6 months postoperative
UPDRS II score	Medication off	21.43 ± 9.00	10.97 ± 6.25^*∗*^	6.81 ± 4.00^*∗*^
UPDRS II score	Medication on	12.60 ± 7.62	8.06 ± 4.66^*∗*^	3.62 ± 2.97^*∗*^
UPDRS III score	Medication off	49.90 ± 12.40	19.38 ± 10.90^*∗*^	14.04 ± 7.73^*∗*^
UPDRS III score	Medication on	27.30 ± 12.16	15.08 ± 9.04^*∗*^	8.40 ± 5.93^*∗*^
LEDD (mg)	—	993.69 ± 505.62	793.52 ± 380.73^*∗*^	477.49 ± 97.44^*∗*^

^*∗*^Compared with preoperative. UPDRS, Unified Parkinson's disease rating scale; LEDD, levodopa equivalent daily dose.

**Table 2:** Baseline characteristics of patients between the two groups.

Variables	Non-POD (*n* = 98)	POD (*n* = 27)>	Total (*n* = 125)	*χ* ^2^/*t*/*Z*	*P*
Male, *n* (%)	43 (431.9%)	9 (33.3%)	52 (41.6%)	0.969	0.325
Age (years)	57.72 ± 9.42	65.55 ± 6.48	59.42 ± 9.41	−4.059	<0.001
BMI (kg/m^2^)	24.15 ± 4.33	23.31 ± 3.76	23.97 ± 4.21	0.915	0.362
Education (years)	11.40 ± 2.78	6.89 ± 3.13	10.42 ± 3.40	7.261	<0.001
ASA grade I	38 (38.8%)	7 (25.9%)	45 (36.0%)	1.596	0.450
II	56 (57.1%)	19 (70.4%)	75 (60.0%)		
III	4 (4.1%)	1 (3.7%)	5 (4.0%)		
Hypertension, *n* (%)	7 (7.1%)	1 (3.7%)	8 (6.4%)	0.418	0.518
Diabetes, *n* (%)	5 (5.1%)	1 (3.7%)	6 (4.8%)	0.091	0.763
Coronary artery disease, *n* (%)	3 (3.1%)	0 (0%)	3 (2.4%)	0.847	0.357
Coronary artery disease, *n* (%)	3 (3.1%)	0 (0%)	3 (2.4%)	0.847	0.357
LEDD (mg)	926.67 ± 464.92	1012.16 ± 516.99	993.69 ± 505.62	−0.777	0.439
Dopamine receptor agonists, *n* (%)	57 (58.2%)	15 (55.6%)	72 (57.6%)	0.059	0.808
MAO inhibitors, *n* (%)	30 (34.1%)	8 (29.6%)	38 (30.4%)	0.186	0.666
Operation time (min)	128.27 ± 48.78	129.74 ± 69.79	128.58 ± 53.69	−0.126	0.900
Anesthesia time (min)	66.00 (20.00)	80.00 (40.00)	70.00 (20.00)	−2.927	0.003
General anesthesia maintenance method					
TIVA	49 (50.0%)	14 (51.9%)	65 (52.0%)	0.547	0.460
CIIA	49 (50.0%)	13 (48.1%)	60 (48.0%)		

BMI, body mass index; ASA, American Society of Anesthesiologists; LEDD, levodopa equivalent daily dose; MAO, monamine oxidases; TIVA, total intravenous anesthesia; CIIA, total intravenous anesthesia.

**Table 3:** Comparison of preoperative cognitive status between the two groups.

Variables	Non-POD (*n* = 98)	POD (*n* = 27)	Total (*n* = 125)	*χ* ^2^/*t*/*Z*	*P*
CDR score	0.00 (0.00)	0.50 (0.00)	0.00 (0.00)	−7.557	<0.001
IADL score	20.00 (1.25)	21.00 (5.00)	20.00 (3.00)	−2.554	0.011
MMSE score	27.67 ± 2.13	23.52 ± 4.00	26.78 ± 3.14	7.237	<0.001
MoCA score	22.94 ± 4.84	17.89 ± 4.71	21.85 ± 5.23	4.830	<0.001
HAMA score	4.18 ± 5.09	8.15 ± 5.55	5.04 ± 5.42	−3.515	0.001
HAMD score	2.50 (8.00)	6.00 (6.00)	5.00 (8.00)	−2.680	0.007
Correct elimination (*n*)	31.00 (2.00)	30.00 (4.00)	31.00 (2.50)	−1.206	0.228
Missing elimination (*n*)	1.00 (2.00)	3.00 (8.00)	1.00 (3.00)	−2.515	0.012
Incorrect elimination (*n*)	0.00 (0.00)	1.00 (5.00)	0.00 (1.00)	−3.514	<0.001
Elimination time (s)	180.80 ± 65.34	215.39 ± 95.88	188.27 ± 73.90	−2.186	0.031
Elimination index (%)	0.53 ± 0.18	0.46 ± 0.16	0.52 ± 0.18	1.945	0.054

**Table 4:** Univariate and multivariate logistic regressions.

Predictor variable	Univariate analysis		Multivariate analysis	
	OR (95% CI)	*P*	OR (95% CI)	*P*
Age	1.13 (1.06–1.21)	<0.001	1.14 (1.03–1.26)	0.010
CDR score	39.38 (12.34–125.63)	<0.001	10.57 (2.54–44.00)	0.001
MMSE score	0.65 (0.54–0.77)	<0.001	0.72 (0.56–0.92)	0.008
HAMA score	1.14 (1.05–1.23)	0.001	1.16 (1.02–1.32)	0.020

**Table 5:** Diagnostic analysis of indicators for the prediction of POD.

Variable	AUC	Cutoff	Sensitivity (%)	Specificity (%)	Youden index
Age	0.751	58.5	92.6	52.0	0.446
CDR score	0.848	0.5	77.8	91.8	0.696
MMSE score	0.827	27.5	88.9	62.2	0.511
HAMA score	0.706	12.5	85.2	54.1	0.393

## Data Availability

The data used to support the findings of this study are available from the corresponding author upon request.

## Additional Points

Delirium after implantation of DBS electrodes is common. Age, CDR score, MMSE score, and HAMA score were indicated to be independent influence factors of POD. It is necessary to assess the cognitive status of PD patients before surgery.
